# Recommendation regarding the cranial upper border of level IIb in delineating clinical target volumes (CTV) for nasopharyngeal carcinoma

**DOI:** 10.1186/s13014-020-01720-2

**Published:** 2020-11-23

**Authors:** Lijun Wang, Shengfu Huang, Lanfang Zhang, Xia He, Yatian Liu

**Affiliations:** 1 Department of Radiation Oncology, Jiangsu Cancer Hospital, Jiangsu Institute of Cancer Research, The Affiliated Cancer Hospital of Nanjing Medical University, Nanjing, China; 2 Department of Radiology, Jiangsu Cancer Hospital, Jiangsu Institute of Cancer Research, The Affiliated Cancer Hospital of Nanjing Medical University, Nanjing, China

**Keywords:** Clinical target volume (CTV), Level IIb, Delineation, Nasopharyngeal carcinoma

## Abstract

**Purpose:**

To recommend a cranial border for level IIb in delineating clinical target volumes (CTV) for nasopharyngeal carcinoma (NPC) patients receiving intensity-modulated radiotherapy and to help reach a consensus on contouring level IIb in CTV.

**Methods:**

From 2012 to 2016, 331 nonmetastatic NPC patients treated with IMRT were retrospectively enrolled. Based on the AJCC 8th staging system of NPC, there were 15 stage I, 76 stage II, 103 stage III, and 137 stage IV patients. The distribution of cervical lymph nodes in NPC was assessed based on imaging. Comparisons of the safety and parotid dose parameters between patients with and without a reduction in the size of level IIb were conducted using SPSS 25.0 and R 2.14.2 software.

**Results:**

Metastasis rates in the most commonly involved lymph nodes, the lateral retropharyngeal and IIb nodes, were 82.8% and 64.0%, respectively. Among patients with level IIb involvement, the upper borders of the metastatic nodes were beyond the caudal edge of C1 in 13.7% of cases. The parotid gland D50 and V26 values were significantly reduced after modifying the upper bound of level IIb used to delineate the CTV (*P* = 0.000).

**Conclusion:**

In principle, the upper bound of level IIb should reach the lateral skull base during delineation of the cervical CTV for NPC. To protect the parotid glands, however, individualized reduction of the upper bound of level IIb is recommended for patients who meet certain criteria.

## Introduction

Compared to other head and neck squamous cell carcinomas, nasopharyngeal carcinoma (NPC) has a distinct epidemiology, etiology, and clinical manifestation [1]. Since NPC has the highest incidence of regional lymph node metastasis among head and neck cancers (HNC), the contouring of the clinical target volume (CTV) in the bilateral neck lymphatic drainage areas is very important in intensity-modulated radiation therapy (IMRT) for NPC [2, 3]. CT-based international consensus guidelines for the delineation of neck CTV in node-negative patients were proposed in 2003 [4], and the guidelines were updated in 2013 [5].

However, these guidelines were primarily derived from patients with head and neck squamous cell carcinomas. Given the unique biological behavior of NPC, there is still controversy surrounding the delineation of the neck CTV for NPC. In an attempt to define more suitable cranial boundaries for level IIb in neck CTV for NPC, we conducted this retrospective study and investigated the distribution of and rate of metastasis in high-seated lymph nodes in level IIb.

## Materials and methods

### Patients and pretreatment evaluations

We performed a retrospective study of 331 patients who met the following criteria in our hospital between February 2012 and December 2016. Inclusion criteria were patients with pathologically confirmed NPC, who had not undergone prior treatment, had no evidence of distant metastases, underwent the complete course of radical IMRT, and had full treatment plan data available, including the isodose distribution and dose-volume histogram (DVH). Exclusion criteria included prior or current other malignancy and prior RT, chemotherapy or surgery (except for diagnostic procedures) to treat the primary tumor or lymph nodes.

The routine workup comprised a complete medical history, physical and neurologic examinations, hematology studies and biochemistry profiles. MRI scans of the head and neck were performed to evaluate the extent of the locoregional disease. Chest and abdominal CT scans and bone scintigraphy were performed to exclude distant metastasis. Medical records and imaging studies were analyzed retrospectively. All patients were restaged according to the 8th edition of the American Joint Committee on Cancer (AJCC) staging system for NPC.

### MR scanning protocol

All MR images were acquired on the same 1.5 T unit (Achieva, Philips, Best, Netherlands) using a head and neck coil. The plain scanning examination was performed to acquire the following sequences: axial/coronal view: T1-weighted, short-term inversion recovery with T2-weighted fat suppression; sagittal view: T1- and T2-weighted imaging; slice thickness = 5 mm and spacing = 1 mm. Enhanced scanning was performed as follows: axial, coronal, and sagittal fat-suppressed T1-weighted imaging after intravenous injection of 0.1 mmol/kg Gd-DTPA (Bayer Pharma AG, Leverkusen, Germany).

### Image assessment

All MR scans were evaluated by a multidisciplinary NPC treatment group that included three radiation oncologists and two diagnostic radiologists; all disagreements were resolved by consensus. Radiologic criteria for the diagnosis of lymph node metastasis were based on the literature.

The diagnostic criteria for retropharyngeal lymph node (RPLN) and cervical lymph node (CLN) involvement included the following [6–10]: (1) any visible LN in the median RPLNs; a shortest axial dimension of ≥ 5 mm in the lateral RPLNs, of ≥ 11 mm in the jugulodigastric region or of ≥ 10 mm in other cervical regions; or a group of three LNs that were borderline in size; or (2) LNs of any size demonstrating evidence of necrosis or extracapsular spread (ES). The definition of central necrosis on MRI was a focal area of high signal intensity on T2-weighted images or a focal area of low signal intensity on T1-weighted images, with or without a surrounding rim of enhancement. The criteria for ES were the presence of indistinct LN margins, irregular LN capsular enhancement, or infiltration into the adjacent fat or muscle. Lymph node locations were based on the International Consensus Guidelines for neck level delineation [5].

### Treatment

All patients received IMRT. Patients were immobilized in the supine position with a thermoplastic mask. Target volumes were defined by ICRU50 and 62 (International Commission on Radiation Units and Measurements) [11, 12]. The gross tumor volume (GTV) included the primary tumor (GTV-T) and metastatic lymph nodes (GTV-N). CTV-1 (defined as the high-risk clinical target volume) should include the GTV plus a 5- to 10-mm margin and cover the entire nasopharynx, parapharyngeal space, and retropharyngeal nodal regions. CTV-2 (defined as the low-risk clinical target volume) included CTV-1 plus a 5 mm margin and encompassed the maxillary sinus (limited to 5 mm anterior to the posterior nasal aperture and maxillary mucosa), pterygopalatine fossa, posterior ethmoid sinus, parapharyngeal space, skull base, the anterior third of the clivus and cervical vertebra, inferior sphenoid sinus, and cavernous sinus. CTV-N (the clinical target volume of the neck nodal regions) included bilateral coverage of levels II, III, IV, and V, which were outlined according to the recommendations from the Radiation Therapy Oncology Group (RTOG)/European Organisation for Research and Treatment of Cancer (EORTC) consensus delineations for head and neck malignancies [3, 5]. The selection of level IIb contouring methods is detailed below. Radiation was delivered using a simultaneous integrated boost-IMRT technique. The prescribed radiation dose was gradated as follows: a total dose of 66–70 Gy in fractions at 2.18 Gy/fraction was delivered to GTV-P and GTV-N, 60 Gy at 1.875 Gy/fraction was delivered to CTV-1, 50.4 Gy at 1.8 Gy/fraction was delivered to CTV-2, and 50.4–60 Gy was delivered to CTV-N in 28–32 fractions. The normal tissue constraints and plan evaluation were performed following the RTOG 0225 protocol [13]. A total of 282 patients received 1–2 cycles of cisplatin-based chemotherapy, and 26 received 3–4 cycles. Whenever possible, salvage treatments (including boost irradiation, re-IMRT, surgery, and chemotherapy) were provided for patients who developed relapse or persistent disease.

### Selection of level IIb contouring methods

Two methods were used to contour the level IIb region. The first delineated the cranial border of level IIb to the skull base, according to the RTOG 0615 guidelines (control group) [14]. The other method, which contoured the cranial border of level IIb to the lateral process of the atlas, was used in patients who met the following criteria (referred to as the modified group): (1) the primary tumor demonstrated no expansion in the posterior or lateral directions on the ipsilateral side; (2) no positive retropharyngeal LNs (LN_RP_) were present on the ipsilateral side; (3) on the ipsilateral side, the primary tumor did not invade the carotid sheath area, or did invade the carotid sheath area but demonstrated < 90° of invasion (the degree of contact arch between the tumor and carotid artery was less than 90°); (4) there was no positive lymph node in level II above the cranial edge of the second cervical vertebra (C2); (5) there was no visible lymph node in level II from the skull base to the upper edge of C2.

### Follow-up

Follow-up data was measured from the first day of treatment to the day of last examination or death. Patients underwent weekly physicals and hematology-related examinations during the radiotherapy process. The follow-ups were conducted every 3–4 months during the first 2 years, then every 6–12 months from year 3 to year 5 after radiotherapy. Follow-up examinations included a complete physical examination, blood tests, standard nasopharyngeal MRI scan, chest and upper abdominal enhanced CT scan (or chest radiography and abdominal ultrasound), bone scan, and fiber nasopharyngoscopy.

Last follow-up date is Dec 4th, 2018. Primary outcomes are LCR and DFS, and secondary outcomes include OS, DMFS, and parotid gland radiation dose parameters.

### Statistical analysis

SPSS version 25.0 (SPSS Inc., Chicago, IL) and R version 3.0.2 (www.r-project.org) were used for data analysis. The Kaplan–Meier method was used for survival analysis, and log-rank tests were applied to compare differences. χ^2^ tests were used to compare categorical variables, and independent *t *tests were used to compare the means of continuous variables.

To balance the distribution of baseline characteristics, we used propensity score matching (PSM). PSM was performed via logistic regression analysis and included age, gender, AJCC staging, AJCC T classification, AJCC N classification and chemotherapy status. Patients were matched 1:1 based on their propensity scores. All statistical tests were 2-tailed, with the significance level set at < 0.05.

## Results

### Incidence and distribution of nodal metastasis

A total of 295 (89.12%) cases had involved lymph nodes, and 135 (40.79%) had bilateral lymph node metastasis. Retropharyngeal LNs and level IIb LNs were the most commonly involved lymph nodes, with metastasis rates of 82.8% and 64%, respectively. The distribution is detailed in Table 1.

**Table 1:** Detailed distribution of the 331 cases with involved lymph nodes

	Unilateral	Bilateral	Total percentage (%)
Rouviere's LN	125	149	82.8
Medial group of pharynx LN	1	0	0.3
Level Ib	2	5	2.1
Level IIa	105	30	40.8
Level IIb	144	68	64.0
Level III	101	14	34.7
Level IVa	33	1	10.3
Level IVb	1	0	0.3
Level Va	35	2	11.2
Level Vb	7	2	2.7
Level Vc	3	0	0.9
Parotid LN	12	0	3.6

### Distribution of nodal in perch of level II

**Fig. 1:**
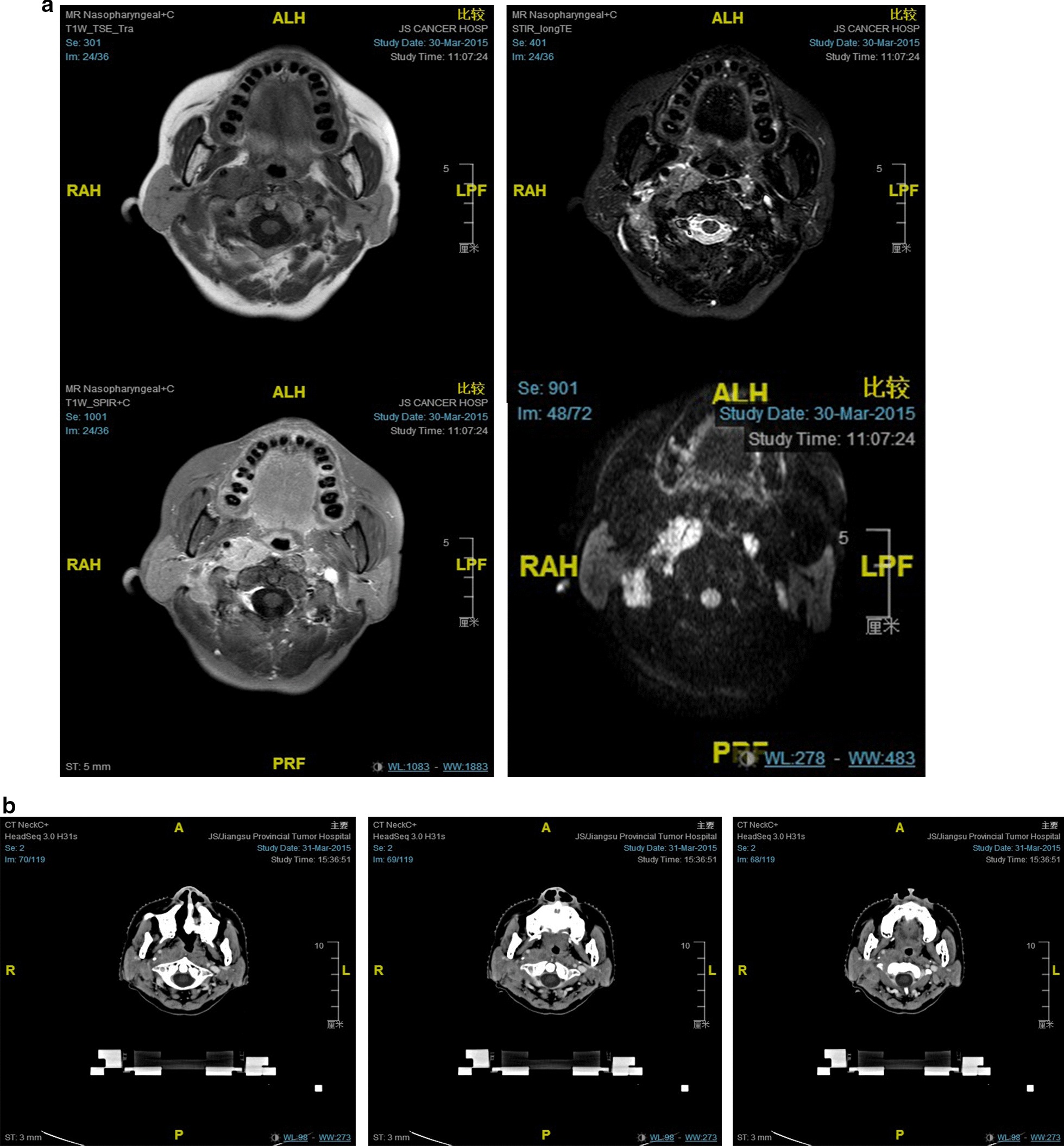
A case with an involved lymph node superior to the upper border of level IIb. **a** MRI (T1-weighted, T2-weighted, T1 C+ and DWI). **b** Three successive slides from a planning CT

Of the 212 patients with level IIb node involvement, the upper border of the metastatic lymph nodes relative to the cervical vertebra was assessed. It was found that the upper border in 58.02% (123/212) of the patients was reaching the cephalic edge of the second cervical vertebra (C2), it exceeded the caudal edge of the lateral process of the first vertebra (C1), the suggested upper border of level II in the 2013 updated international consensus guidelines, in 13.2% (28/212), and it reached the skull base in 2.83% (6/212) (Fig. 1). The distribution of the upper lymph nodes in level II is shown in Table 2.

**Table 2:** Distribution of upper lymph nodes in level IIb

Level IIb	Number (percentage) of patients			
	LNc2		LNab	
	Unilateral	Bilateral	Unilateral	Bilateral
Positive LNs	99 (29.9)	24 (7.25)	26 (7.85)	2 (0.60)
LN ≥ 5 mm	24 (7.25)	13 (3.93)	1 (0.3)	0 (0)

LNc2: level IIb LNs located above the C2 vertebra; LNab: LNs above the upper border of level IIb

However, all of the 135 cases with level IIa node involvement had upper lymph node borders below the caudal edge of the lateral process of C1.

### Optimization of the level IIb contouring method

As mentioned above, two methods were used to contour level IIb based on the different MR imaging characteristics of the tumors. A total of 124 patients were included in the control group, which delineated the cranial border of level IIb to the skull base, according to the RTOG 0615 guidelines. The other 207 cases, which met the optimization criteria mentioned above, were included in the modified group. In the modified group, the upper border of level IIb in the cervical CTV was contoured to the lateral process of C1.

### Propensity score-based survival analysis

The baseline patient characteristics of the control group and the modified group were significantly different (Table 3). To balance the distribution of baseline characteristics, we used propensity score matching (PSM). Following PSM, baseline characteristics between the two groups were similar (Table 3). We collected 103 matched pairs and compared the 5-year overall survival (OS), 5-year local control rate (LCR), 5-year distant metastasis-free survival (DMFS) and 5-year disease-free survival (DFS) between the two groups.

**Table 3:** Characteristics of patients included in the study

Characteristic	Before matching			Case–control		
	Modified group	Control group	*p *value	Modified group	Control group	*p *value
	N = 207	N = 124		N = 103	N = 103	
Age, mean (SD), years	49.9 (11.48)	45.7 (14.57)	0.007	48.25 (13.24)	47.79 (13.58)	0.803
Age, No (%)			0.003			0.385
≥ 46 years	146 (70.5)	68 (54.8)		69 (67.0)	62 (60.2)	
< 46 years	61 (29.5)	56 (45.2)		34 (33.0)	41 (39.8)	
Gender, No (%)			0.030			1.000
Male	161 (77.8)	84 (67.7)		73 (70.9)	73 (70.9)	
Female	46 (22.2)	40 (32.3)		30 (29.1)	30 (29.1)	
AJCC staging^a^, No (%)			0.014			1.000
I–II	66 (31.9)	25 (20.2)		21 (20.4)	21 (20.4)	
III–IV	141 (68.1)	99 (79.8)		82 (79.6)	82 (79.6)	
AJCC T classification^a^, No (%)			0.777			0.843
T1	59 (28.5)	35 (28.2)		23 (22.3)	28 (27.2)	
T2	35 (16.9)	18 (14.5)		14 (13.6)	14 (13.6)	
T3	49 (23.7)	31 (25.0)		26 (25.2)	26 (25.2)	
T4	64 (30.9)	40 (32.3)		40 (38.8)	35 (34.0)	
AJCC N classification^a^, No (%)			0.000			0.107
N0	31 (15.0)	5 (4.0)		9 (8.7)	4 (3.9)	
N1	111 (53.6)	58 (46.8)		56 (54.4)	50 (48.5)	
N2	49 (23.7)	34 (27.4)		28 (27.2)	28 (27.2)	
N3	16 (7.7)	27 (21.8)		10 (9.7)	21 (20.4)	
Chemotherapy, No (%)			0.013			1.000
No	5 (4.0)	18 (8.7)		4 (3.9)	4 (3.9)	
Yes, 1–2 cycles	103 (83.1)	179 (86.5)		90 (87.4)	90 (87.4)	
Yes, 3–4 cycles	16 (12.9)	10 (4.8)		9 (8.7)	9 (8.7)	

^a^Defined by the AJCC 8th staging system criteria

**Fig. 2:**
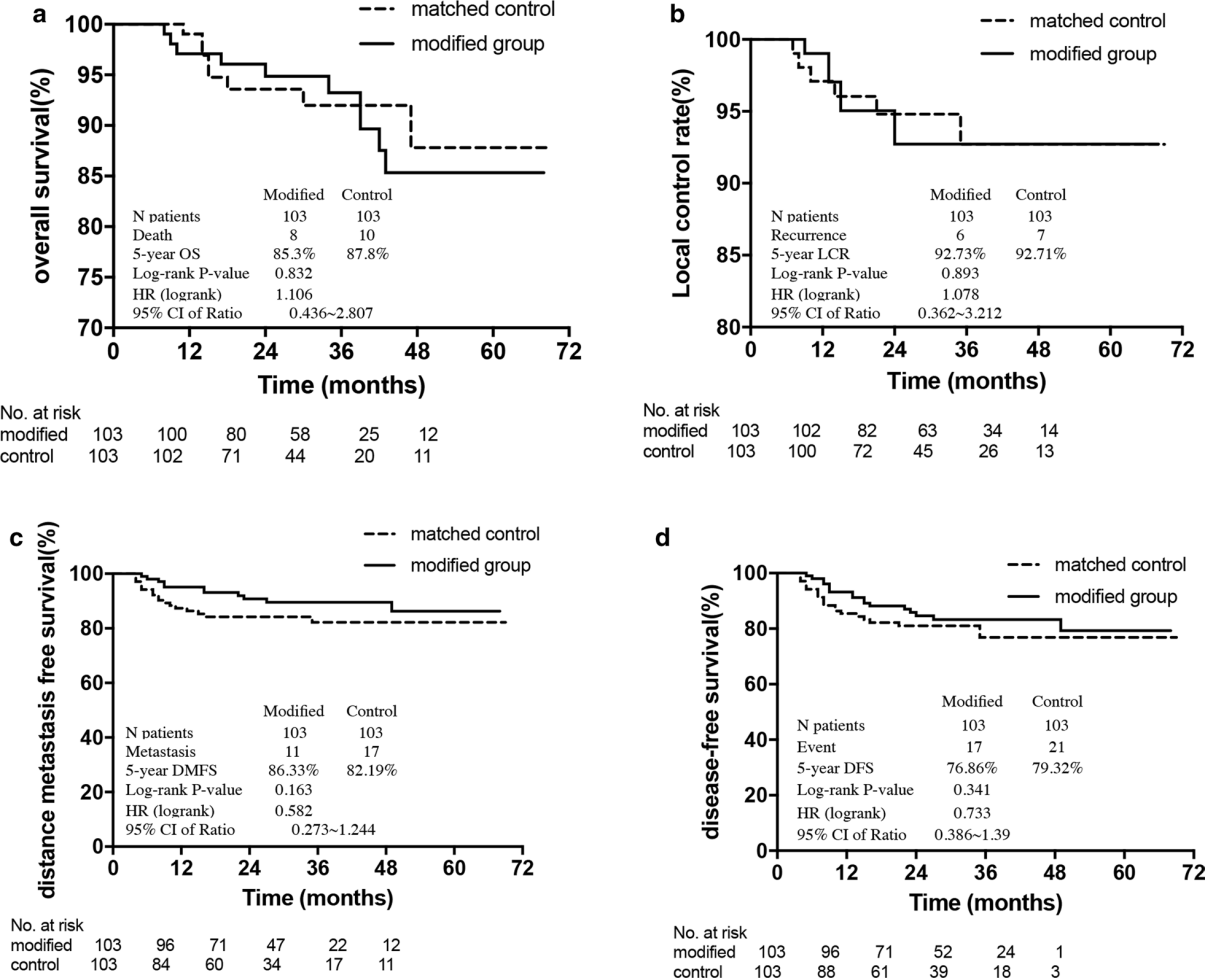
PSM-based survival curves of overall survival (**a**), local control (**b**), distant metastasis-free survival (**c**), and disease-free survival (**d**) between the modified and control groups in the 206 patients with NPC

Kaplan–Meier curves display the OS, LCR, DMFS and DFS results in the modified and control groups (Fig. 2). The 5-year OS, LCR, DMFS and DFS results for the modified group versus control group were 85.3% versus 87.8%, 92.73% versus 92.71%, 86.33% versus 82.19%, and 76.86% versus 79.32, respectively. As shown in Fig. 2, there were no significant differences between the two groups.

### Comparison of parotid gland radiation dose parameters

Level IIb is adjacent to the deep lobe of the parotid gland. In the modified group, the optimized cervical CTV contouring method inevitably reduced the radiation dose delivered to the parotid gland. We compared the parotid gland D50 (the dose received by 50% of the volume) and V26 (the volume receiving 26 Gy) between the two groups. The parotid D50 and V26 were significantly lower in the modified group than in the control group (Table 4).

**Table 4:** Comparison of radiation dosimetric parameters of the parotid glands in the 331 cases

	Group		*p *value
	Modified group	Control group	
Parotid D50 (Gy)			
Left	26.88 ± 5.32	31.77 ± 8.18	0.000
Right	25.99 ± 5.13	31.42 ± 7.54	0.000
Parotid V26 (%)			
Left	52.42 ± 15.94	64.21 ± 17.45	0.000
Right	49.41 ± 14.51	63.79 ± 16.41	0.000

## Discussion

**Fig. 3:**
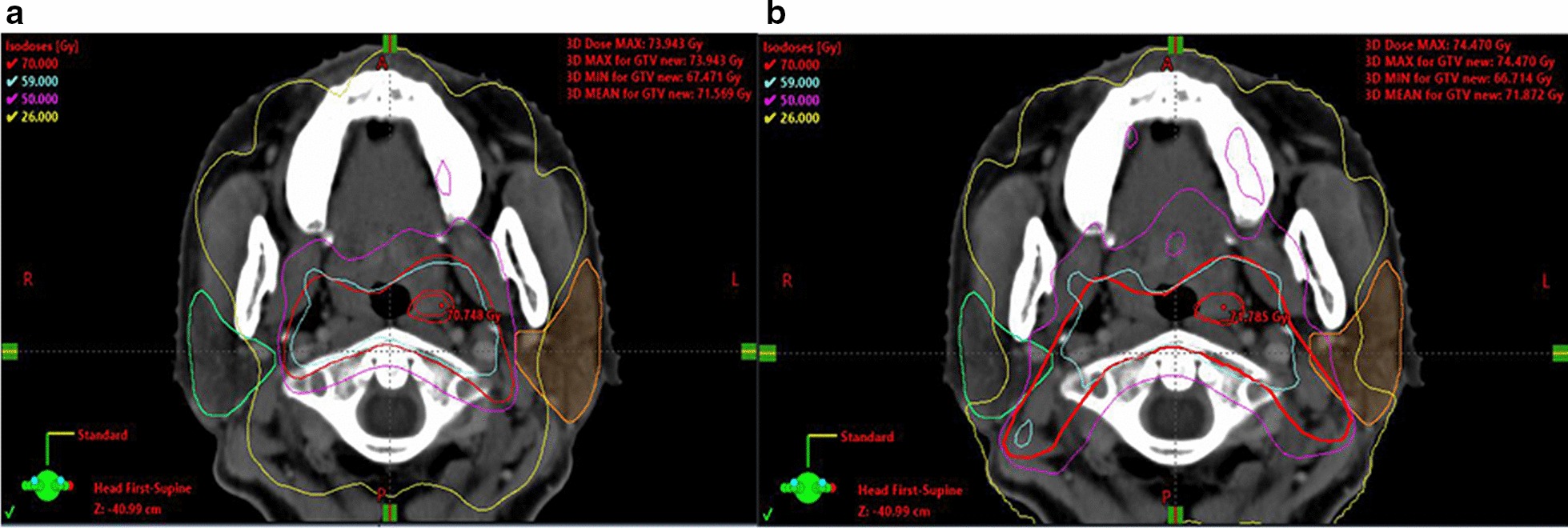
Isodose curve of the same patient. As shown in the figure, the yellow line is the 26 Gy isodose line. Compared with the lateral skull base being included in the CTV (red line) range (**b**) (contouring method in control group), when the CTV does not delineate the lateral skull base (**a**) (contouring method in modified group), the dose delivered to the parotid gland is lower

Since cervical node metastasis is very common in patients with NPC, it is always recommended that the bilateral neck lymphatic drainage areas be irradiated to achieve a higher locoregional control rate, regardless of the stage at presentation [3]. Lymph node metastasis in NPC follows an orderly pattern. In this study, we retrospectively analyzed the distribution of involved lymph nodes in 331 patients with NPC and confirmed that the most commonly involved regions include the retropharyngeal and level IIb lymph nodes, with metastasis rates of 82.8% and 64.0%, respectively (Fig. 3).

In the past two decades, recommendations regarding the selection and delineation of the neck node CTV have been proposed by several researchers [2, 3, 15]. The recently updated guidelines defined in 2013 have proven to be sufficiently comprehensive, and the boundaries described therein are applicable for most levels in cases of standard NPC [2, 16]. However, there is a paucity of knowledge regarding the patterns of NPC nodal metastasis provided by these guidelines. Moreover, there is no evidence that the boundaries in these guidelines fully cover the lymphatic drainage pathway of NPC. Our study reports the distribution of cervical metastatic lymph nodes, and we investigated whether the upper border of level IIb suggested in the new guidelines is sufficiently extensive. The caudal edge of the lateral process of C1 is still the proposed upper border of level II in the new guidelines. Our study showed that among the 212 cases with level IIb nodal involvement, the uppermost border in 28 cases (13.2%) was beyond the proposed boundary, with 123 cases (58.02%) extending beyond the upper margin of C2, and 6 cases (2.83%) reaching the skull base. However, all of the 135 cases with level IIa node involvement were within the proposed boundaries. Zhang et al. [17] and Wang et al. [16, 18] reported that the cranial edge of level II did not fully cover all level II involvement. Hence, some researchers proposed that the upper border of level II should be extended to the skull base for NPC cases, regardless of nodal status. However, consensus regarding this suggestion is low at 64% in agreement [3].

Based on our data, as the cranial edge of level IIb proposed in the updated guidelines did not fully cover all level IIb involvement in a subset of patients, we agree with the suggestion that the upper border of level IIb should be extended up to the skull base for NPC cases in principle. However, the upper border of level IIb could be reduced to the caudal edge of the lateral process of C1 for patients who meet the optimization criteria mentioned above. In the modified group, our CTV was based on the following: (1) high-resolution planning CT, high-quality MRI, and PET-CT (for some patients), (2) the individual tumor extent, and (3) the distinctive orderly and stepwise pattern of spread of NPC. In this study, after case–control matching via PSM, we did not observe significant differences between the two groups with regard to 5-year OS, LCR, DMFS and DFS. Importantly, with a median follow-up of 35 months, there was no marginal or out-of-field recurrence observed with our optimized contouring method of level IIb in neck CTV.

When the upper border of level II in neck CTV is extended to the skull base, the surrounding normal tissues are exposed to increased radiation. In the long term, decreased doses of radiation therapy delivered to the parotid glands should decrease the incidence of xerostomia, which remains high in the IMRT era [19, 20]. Eisbruch et al. [21] and Pointreau et al. [19] proposed that when the radiation dose delivered to the parotid gland is less than 26 Gy, its function would gradually be restored following radiotherapy. Chao et al. [22] predicted that the dose threshold resulting in salivary flow reduction (< 25% of the pretreatment level of stimulated parotid secretion) was 32 Gy. We estimated the parotid 50% volume dose, the D50, and the volume percentage dose at 26 Gy, V26, in both groups. Our results show that the parotid D50 and V26 were markedly lower in the modified group than in the control group.

There are several important limitations to our study. Although all patients were treated using a protocol-based target volume, the study was retrospective in nature. Additionally, there were variations in treatment modality, radiation therapy dosing, and the number of cycles of chemotherapy within our cohort. We overcame this shortcoming by including consecutive patients who were treated at a single center, performing an in-depth review of the imaging studies and medical records, and providing continuous, long-term follow-up. Our results should be validated in further prospective trial.

## Conclusion

In summary, based on our data on the distribution of involved lymph nodes in NPC patients, we agree with the suggestion that the upper bound of level IIb should reach the lateral skull base during the delineation of the neck CTV in principle. To protect the parotid glands, however, individualized reductions of the upper bound of level IIb are recommended for patients who meet the following criteria: (1) the primary tumor demonstrates no expansion in the posterior or lateral directions on the ipsilateral side; (2) no positive retropharyngeal LNs (LN_RP_) are present on the ipsilateral side; (3) on the ipsilateral side, the primary tumor does not invade the carotid sheath area, or does invade the carotid sheath area but demonstrates < 90° of invasion (the degree of contact arch between the tumor and carotid artery is less than 90°); (4) there is no positive lymph node in level II above the cranial edge of the second cervical vertebra (C2); (5) there is no visible lymph node in level II from the skull base to the upper edge of C2.

We did not observe an increase in the rate of local or regional recurrence. The selectively reduced CTV might effectively avoid unnecessary radiation to parotid glands, thereby decreasing the incidence of xerostomia.

## Abbreviations

CTV: Clinical target volumes
NPC: Nasopharyngeal carcinoma
D50: Dose received by 50% of volume
V26: Volume percentage of received 26 Gy
HNC: Head and neck cancer
IMRT: Intensity-modulated radiation therapy
DVH: Dose-volume histogram
MRI/MR: Magnetic resonance imaging
CT: Computed tomography
AJCC: The American Joint Committee on Cancer
RPLN: Retropharyngeal lymph node
CLN: Cervical lymph node
ES: Extracapsular spread
GTV: Gross tumor volume
RTOG: The radiation therapy oncology group
EORTC: European Organisation for Research and Treatment of Cancer
C2: The second cervical vertebra
PSM: Propensity score matching
OS: Overall survival
LCR: Local control rate
DMFS: Distant metastasis-free survival
DFS: Disease-free survival
LNc2: Level IIb LNs located above the C2 vertebra
LNab: LNs above the upper border of level IIb

## Availability of data materials

The datasets used and/or analyzed during the current study are available from the corresponding author on reasonable request.
